# Composition and Acute Inflammatory Response from *Tetraponera rufonigra* Venom on RAW 264.7 Macrophage Cells

**DOI:** 10.3390/toxins13040257

**Published:** 2021-04-03

**Authors:** Suwatjanee Naephrai, Supakit Khacha-ananda, Pornsiri Pitchakarn, Churdsak Jaikang

**Affiliations:** 1 Toxicology Section, Department of Forensic Medicine, Faculty of Medicine, Chiang Mai University, Chiang Mai 50200, Thailand; Suwatjanee_n@cmu.ac.th (S.N.); supakit.kh@cmu.ac.th (S.K.-a.); 2 Department of Biochemistry, Faculty of Medicine, Chiang Mai University, Chiang Mai 50200, Thailand; pornsiri.p@cmu.ac.th

**Keywords:** inflammation, *COX-2/mPGES-1* pathway, hymenoptera

## Abstract

*Tetraponera rufonigra* (Arboreal Bicoloured Ant) venom induces pain, inflammation, and anaphylaxis in people and has an increased incident in Southeast Asia regions. The bioactive components and mechanism of action of the ant venom are still limited. The aim of this research was to identify the protein composition and inflammatory process of the ant venom by using RAW 264.7 macrophage cells. The major venom proteins are composed of 5’ nucleotidase, prolyl endopeptidase-like, aminopeptidase N, trypsin-3, venom protein, and phospholipase A_2_ (PLA_2_). The venom showed PLA_2_ activity and represented 0.46 μg of PLA_2_ bee venom equivalent/μg crude venom protein. The venom induced cytotoxic in a dose- and time-dependent manner with IC_20_ approximately at 4.01 µg/mL. The increased levels of COX-2 and PGE_2_ were observed after 1 h of treatment correlating with an upregulation of *COX-2* expression. Moreover, the level of *mPGES-1* expression was obviously increased after 12 h of venom induction. Hence, our results suggested that the induction of *COX-2/mPGEs-1* pathway could be a direct pathway for the ant venom-induced inflammation.

## 1. Introduction

*Tetraponera rufonigra*, an arboreal bicolor ant, is one of the most dangerous invasive pests on a global scale [1]. *T. rufonigra* is classified as the Hymenoptera, family Formicidae, and subfamily Psudomyrmecinae. It can be found in widespread regions in Pakistan, India, and Southeast Asia [1,2]. This ant species is important in medicine since the ant venoms have induced an anaphylaxtic reaction [2]. Clinical manifestations including pain, urticarial, angioedema, dyspnea, and loss of consciousness have been reported in patients who were exposed to the ant venom [3].

Normally, the ant venom consists of various types of proteins, alkaloids, hydrocarbons, and formic acid [4,5,6]. These substances exerted paralytic, cytolytic, hemolytic, and allergenic effects after exposure [4,5]. Protein is a major component in ant toxin such as, phospholipase A_1_ (PLA_1_), phospholipase A_2_ (PLA_2_) [7], antigen 5 [8], and metalloproteinase which damage the tissue and induce inflammation. The investigation of venom composition in genus *Tetraponera* has been identified [9,10]. It contained pseudomyrmecitoxins (PSDTX), phospholipase, and venom allergens which acted as a defensive venom with a very painful action to aggressors. In addition, the mixture of eight alkaloids especially tetraponerine-8 was discovered in *Tetraponera sp*. which was classified to be a toxic chemical defense against other ant species [9,10]. The venom protein of Fierce Stinging Ant (*Tetraponera aethiops*) shared the identity sequence with the venom protein from *Odontomachus monticola, Myrmecia pilosula, Myrmecia gulosa*, and *Tetramorium bicarinatum*. The most abundant venom protein in the Fierce Stinging Ant was identified as phospholipases and venom allergen [9].

Inflammation is a host response after exposure to the ant venom. Many classes of white blood cells such as macrophage and neutrophil were activated to release inflamma-tory mediators including prostaglandins, pro-inflammatory cytokines, and chemokine [11,12,13]. Prostaglandins and lipid products are involved in both biological and physical processes including blood pressure, smooth muscle contraction, bronchoconstriction, pulmonary vasoconstriction, peripheral vasodilation [14,15], and allergic symptoms [16]. Prostaglandins are produced by the conversion of arachidonic acid to prostaglandin G_2_ (PGG_2_) using the cyclooxygenase-1 and -2 enzymes (COX-1 and COX-2), followed by further processing with prostaglandin synthases. Prostaglandin E_2_ (PGE_2_) is a pro-inflammatory and immunomodulatory lipid mediator formed from PGH_2_ by microsomal prostaglandin E synthase 1 (mPGES-1) which is an inducible inflammatory enzyme and linked to pathophysiological conditions, such as inflammation, pain, fever, and tumorigenesis [17,18].

Many publications have represented the ability of hymenoptera venom to induce *COX-2* gene and protein expressions. An increase in the pro-inflammatory cytokine levels such as IL-1, 6, 8, 12, and TNF-α was observed after exposure of the *Dinoponera quadriceps* and *Neoponera villosa* venom in RAW 264.7 macrophage cells [19,20]. Additionally, some publications demonstrated that the P17, an original host defense peptide from the ant venom induces antifungal activities of macrophages through the induction of C-Type Lectin receptors [21].

The PLA_2_ enzyme is widely distributed in the hymenoptera venom and can cleave a phospholipid membrane at the sn-2 position resulting in the release of arachidonic acid and lysophospholipid [4,5,7,22]. Ant venom proteins are caused by paralytic, cytolytic, and hemolytic responses [4,5,6] and some proteins are allergens, inducers, and promoters of anaphylactic reactions in the victim [1,3]. Previous publications demonstrated that environmental and genetic factors contributed to the chemical composition in the ant venom [1]. The *Ectatomma brunneum* ants collected from different environmental conditions, such as urban, intermediate, wood-land, and monoculture sites significantly showed different chemical profiles of the venoms [23]. Moreover, the dietary and nest site could alter the venom chemical profile [23,24]. The population ecology of *T. rufonigra* was observed in Penang Island, Malaysia, demonstrating a genetic divergence even through studies in nearby locations [25].

Additionally, the difference in environmental and genetic factors contributes to the chemical composition of the ant venom, as described above. Many researchers attempted to discover the chemical composition of several kinds of ants in a specific location. However, the protein composition and specific toxicity of the venom of *T. rufonigra* collected in Northern Thailand remained poorly characterized. Hence, our study aimed to investigate the protein composition and the induction of inflammatory process of *T. rufonigra* venom.

## 2. Results

### 2.1. Ant Characteristics

**Figure 1:**
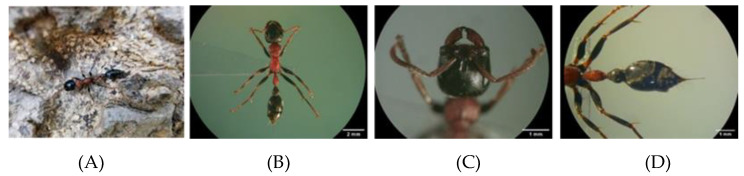
The morphology of *T. rufonigra* under a stereo microscope. The body (**A**), head, and gaster (**B**), mandibles, (**C**) and the gaster (**D**).

The ant specimen was identified by Dr. Weeyawat Jaitrong, a taxonomist at the Natural History Museum, Thailand. The body length was 1.0 to 1.2 cm in size. The head, abdomen, legs, and post petiole of worker ants were black, while thorax was orange-brown to dark-reddish and rough. The body was slender, the color of alitrunk was light orange-brown, and the head and gaster were black. The head was slightly longer than broad with mandibles of five or six teeth. The morphology of *T. rufonigra* is shown in Figure 1.

### 2.2. The Venom Protein Identification

**Figure 2:**
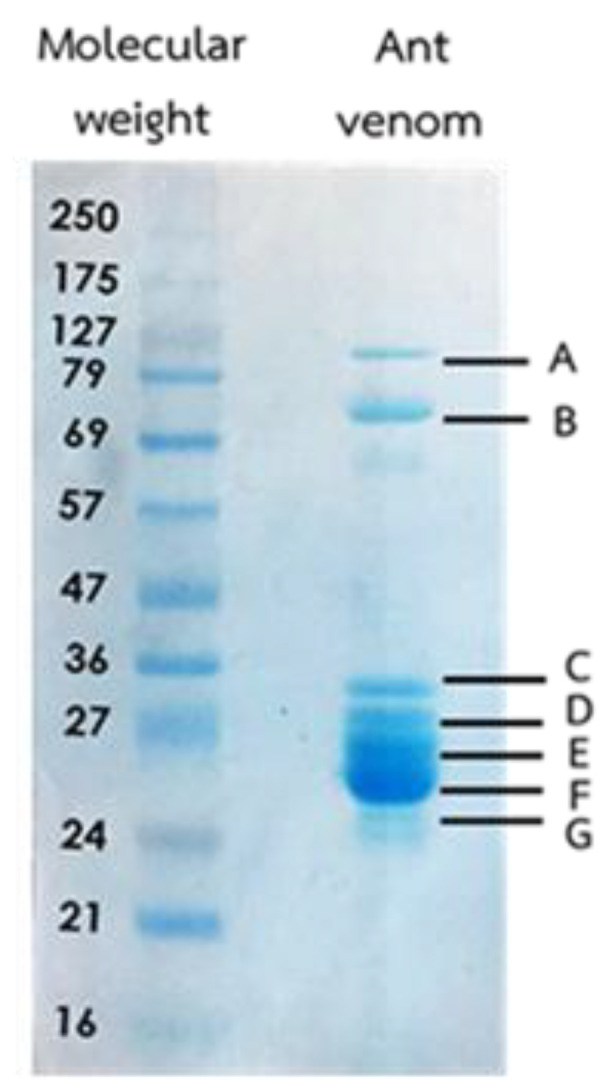
The SDS-PAGE protein separation of *T. rufonigra* venom. The crude venom proteins were run with protein markers ranging from 16–250 kDa at 150 V for 140 min kDa in 15% bis-acrylamide gel and the gels were stained with coomassie blue. The experiments were done in triplicate experiments.

The ant venom was harvested from 1875 worker ants and the protein concentration was measured by the bicinchoninic acid (BCA) protein kit assay. The protein concentration in the crude venom was 206.03 µg/µL. Thirty micrograms of the crude venom were subjected to sodium dodecyl sulfate polyacrylamide gel electrophoresis (SDS-PAGE) for the separation. The major protein bands are represented in Figure 2. There were seven dominant protein bands (A to G) with molecular weight ranging between 24–127 kDa. The protein band between 24–27 kDa presented the most relative abundance with both high intensity of staining and thickness of protein band. All seven dominant protein bands were cut to identify the type of protein by high-performance liquid chromatography/electrospray ionization tandem mass spectrometry (HPLC-ESI-MS/MS).

The type of venom proteins were identified by a sequence alignment with the NCBI database. A total of 17 protein families were identified as shown in Table 1. All venom proteins were separated into seven groups (Table 2) according to their function as the venom protein, the transcription activator/regulation protein, cell cycle control protein, transporter protein, structural protein, ligand protein, and hypothetical protein.

### 2.3. Secreted Phospholipases A_2_ (sPLA_2_) Activity 

**Figure 3:**
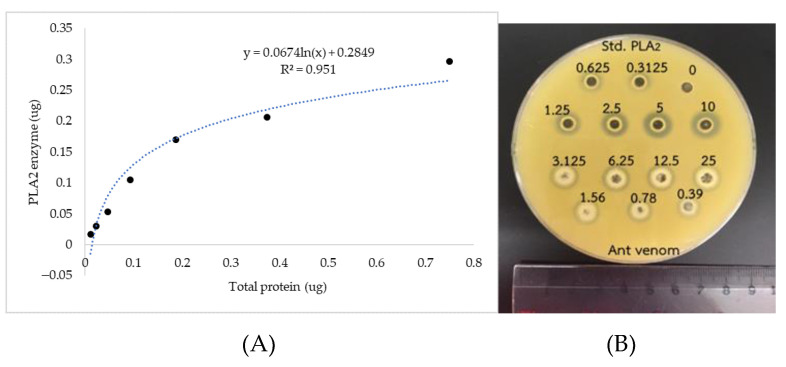
(**A**) The relative amount of PLA_2_ contained in the crude venom protein. (**B**) The clear zone was observed in an egg yolk agar plate after incubating with the standard PLA_2_ from the bee venom and the crude *T. rufonigra* venom protein in the range of 0.39–25 µg/mL at 37 °C for 18 h.

To screen the level of secreted PLA_2_ contained in the ant venom, the agar-plate containing egg yolk phosphatidylcholine (PC) was chosen to detect the venom phospholipase activity. After the ant venom was added into the egg yolk plate, the size of clear zone was observed and measured which was in accordance to the amount of phospholipase A_2_ (Figure 3). The amount of PLA_2_ contained in the ant venom was calculated in comparison with the bee venom PLA_2_ standard curve (0.3125 to 10 µg/mL) and presented 0.46 μg of bee venom phospholipase A_2_ equivalent/μg venom protein. 

### 2.4. RAW 264.7 Macrophage Cell Viability

**Figure 4:**
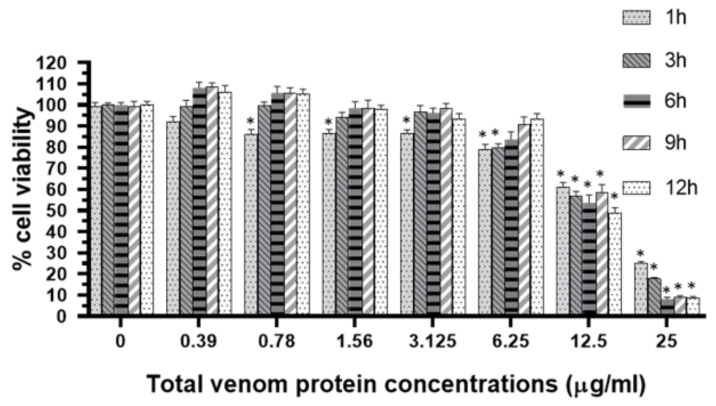
The reduction of cell viability was clearly observed after treating the cells with various concentrations of the ant venom ranging from 0.39 to 25 μg/mL for 1, 3, 6, 9, and 12 h. The cell viability is expressed as the mean ± SEM of triplicate experiments. * *p* < 0.05, a significant difference compared with the control group.

The cytotoxicity of the ant venom on RAW 264.7 cells was determined before the inflammatory assessment. RAW 264.7 cells were treated with various concentrations of the ant venom (0.39–25 µg/mL) for 1, 3, 6, 9, and 12 h and then the MTT assay was performed. The percent of cell viability was dependent on the dose and time after treating the cell with the ant venom. The cell viability tended to increase in a time-dependent manner, whereas the viability of cells treated with the ant venom at concentrations of 12.5 and 25 µg/mL was noticeably reduced (Figure 4). Therefore, the inhibitory concentrations causing 20% (IC_20_) cytotoxicity of approximately 4.01 µg/mL were used for further experiments.

### 2.5. Quantification of COX-2 in Venom-Treated Cells

**Figure 5:**
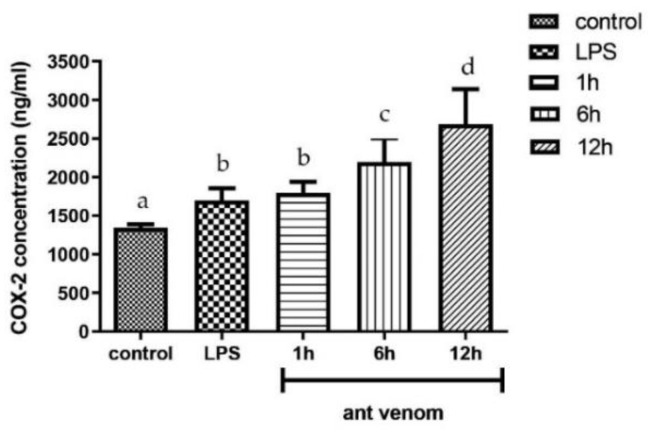
The COX-2 level after treating RAW 264.7 macrophages with the *T. rufonigra* venom at 4.01 µg/mL. The results are presented as the mean ± standard error (SEM). Samples represented with different small letters are significantly different from the other groups (*p* < 0.05).

To quantify the key inflammatory mediator released from ant venom-treated cells, the cells were treated with the IC_20_ concentration of the ant venom. The intracellular COX-2 was determined by the commercial ELISA kit. The COX-2 concentration in lipopolysaccharides (LPS)-treated cells which was used as a positive control was 1644.11 ± 43.29 ng/mL. The COX-2 level of the ant venom-treated cells was significantly increased in a time-dependent manner compared with the control group (*p* < 0.05), as shown in Figure 5. The cells treated with the ant venom for 12 h presented the highest level of intracellular COX-2 production by 2683.32 ± 453.80 µg/mL. 

### 2.6. Measurement of Prostaglandin E2 (PGE_2_)

**Figure 6:**
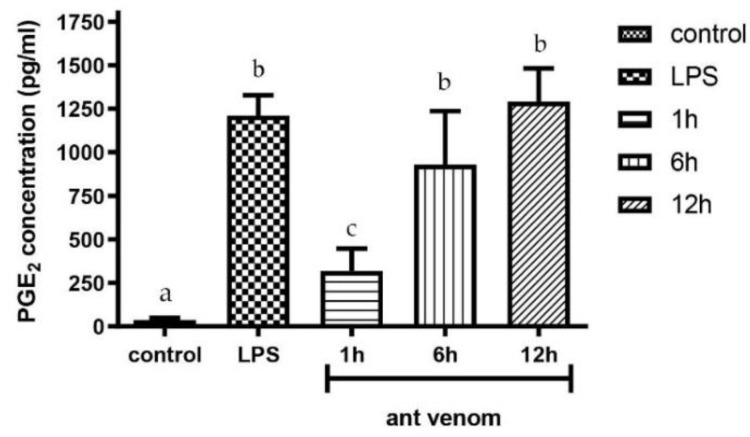
The secreted PGE_2_ level in the culture medium of RAW 264.7 cells treated with the *T. rufonigra* venom at 4.01 µg/mL. The results are presented as the mean ± standard error (SE). Samples represented with different small letters are significantly different from the other groups (*p* < 0.05).

The previous experiment showed the induction of intracellular COX-2 production, which appeared to be the key step of PGE_2_ formation during the inflammatory process. This experiment aimed to determine whether the secreted PGE_2_ in the culture medium of the cells treated with the ant venom increased using the commercial ELISA kit. The secreted PGE_2_ level in the LPS-treated cells (a positive control) was 1211.00 ± 116.51 pg/mL. The amount of the secreted PGE_2_ in the venom treated cells was significantly increased in a time-dependent manner compared to the non-treated control (Figure 6). The cells incubated with the ant venom for 12 h showed the highest level of secreted PGE_2_ by 1292.05 ± 189.87 µg/mL.

### 2.7. The mRNA Expression of COX-2, mPGES-1, and cPLA_2_

**Figure 7:**
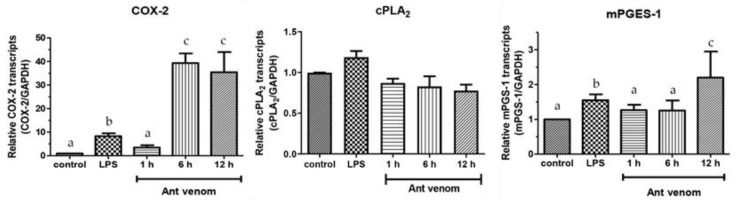
The mRNA expression of *COX-2*, *cPLA_2_*, and *mPGES-1* was induced by the *T. rufonigra* venom in RAW 264.7 macrophage cells at a concentration of 4.01 µg/mL for 1, 6, and 12 h. The lipopolysaccharides (LPS) (1 µg/mL) was used as a positive control. The results are presented as the mean ± standard error (SE). Samples represented with different small letters are significantly different from the other groups (*p* < 0.05).

After an evaluation of COX-2 and PGE_2_ protein levels in macrophages following an in vitro exposure to the ant venom, we determined the mRNA expression of *COX-2*, *mPGES-1,* and *cPLA_2_* expression in the treated cell. The ant venom-treated cells were collected and subjected to total RNA extraction by the nucleozol reagent and RT-qPCR. The results demonstrated that the overexpression of *COX-2* and *mPGES-1*, but not *cPLA_2_* were observed in the ant venom-treated cells. The *COX-2* expression was increased about 3.5-folds after the treatment with the ant venom for 1 h and continuously increased about 27-folds during 6 and 12 h of incubation. The expression of *mPGES-1* gene was significantly upregulated after the treatment for 12 h (Figure 7).

## 3. Discussion

In the present study, the seven dominant protein bands with a molecular weight ranging from 24–127 kDa were discovered in the *T. rufonigra* venom. The molecular weight of the ant venom proteins found in our study was similar to the *Dinoponera quadriceps* venom [2]. In addition, a previous study identified several ant venom proteins from other species of ants such as venom protein with a molecular weight ranging from 15.2–70.1 KDa of Solenopsis invicta venom [22], 20.1–97 kDa of *Neoponera villosa* venom [19], 18–160 kDa of *Odontomachus bauri* venom [26], and 12–85 kDa of *Brachyponera chinensis* [26]. The ant venom proteins were identified with LC-MS and classified into seven groups using their functions including venom protein, transcription activator/regulation protein, cell cycle control protein, transporter protein, structural protein, ligand protein, and hypothetical protein. The 5’nucleotidase (5NUC) is a hydrolytic enzyme and can be widely found in venomous animals especially snakes and bees [20,26]. The function of this protein is to release adenosine from nucleotides and adenosine monophosphate resulting in the paralysis of the victim [26]. The peptidase enzymes such as prolyl endopeptidase-like, putative aminopeptidase, aminopeptidase N (APN), trypsin-3, and serine-type endo-peptidase were recently presented in the studied ant venom. The prolyl endopeptidase-like enzyme has been reported in the venom of Hairy Panther Ant (*Neoponera villosa*) [3] and Chinese scorpion (*Mesobuthus martensii*) [27]. The function of this enzyme is to digest internal peptide bonds in the tissue of their prey. Moreover, the APN and trypsin are digestive enzymes that are released for the degradation of prey tissue. They could be found in the predator ant (*Pachycondyla striata*) [28], Endoparasitoid wasp (*Cotesia chilonis*) [29], snake, and bee venom [2]. It was found that the *T. rufonigra* venom protein showed 59% similarity with the venom protein of the parasitoid jewel wasp (*Ampulex compressa*) after alignment with the NCBI database. This protein function was on the paralysis of the central nervous system of victims [30]. A 2D gel electrophoresis should be done to identify several other proteins.

Phospholipase A_2_ (PLA_2_) is an enzyme widely presented in social hymenoptera venom including the bee, wasp, and ant. The PLA_2_ cleavages membrane phospholipid in a sn-2 position lead to releasing free fatty acids and lysophospholipids [7], disrupting cellular membranes, and inducing pore formation. Our study showed that the PLA2 in *T. rufonigra* venom showed a molecular weight in the range of 24–26 kDa which was similar to the South American bullet ant (*Paraponera clavata*) and fire ant (*S. invicta*) venom [4,7]. Regarding the transcription activator/regulation protein group, two proteins, transcriptional activator cubitus interruptus and transcription factor SPT20 homolog isoform X1 were identified. 

Although our studies discovered various kinds of venom proteins, we further focused on the production of phospholipase in the ant venom. The phospholipase is known as an inducer of immunological activation by enhancing an adaptive response of white blood cells. The phospholipase A1, A2, and B have been commonly identified as a common insect venom component [31,32]. During the envenoming process, the insect venom PLA1 and A2 induced several direct toxic effects [31] and induced a hypersensitivity reaction including potentially fatal anaphylaxis [22]. We assumed that the *T. rufonigra* venom probably consisted of the phospholipase. Our result showed that the phospholipaseA_2_ was contained in the *T. rufonigra* venom according to the clear zone appearance in egg yolk agar. Due to the size of clear zone from the *T. rufonigra* venom measured and compared with a standard bee venom, it is clearly visible that the bee venom showed a high toxicity than the *T. rufonigra* venom in the same concentration. The appearance of a clear zone was similar to the LC-MS results that found the phospholipaseA_2_ enzyme in the ant venom. Schmidt et al. 1998 and Chen et al. 1997 suggested that the precipitation zone with an opaque cloudiness and cream-colored precipitate might result from PLB and PLC [33,34]. However, our experiment in 1D gel electrophoresis and HPLC-MS did not reveal the presence of PLB and PLC in the ant venom. Here, it was probably caused from a limitation of this technique. To clarify this issue, the 2D gel electrophoresis which had more resolution should be done in the next study.

In addition, the investigation of the ant venom toxicity was evaluated. The cell viability of the ant venom-exposed RAW 264.7 cells was decreased in dose- and time-dependent manners. The significant reduction of the cell viability was observed after incubating the cells with the ant venom at high concentrations. It was likely that the mixture of chemical substances such as phospholipase, trypsin-3, and prolyl endopeptidase-like enzyme in the ant venom affected the cell viability via cell membrane disruption, integral protein membrane cleavage, and degradation of neural cell adhesion molecules [22,35]. The immunomodulation of the ant venom at a low concentration should be further investigated. Our results demonstrated that the *COX-2* gene expression tended to increase in the first hour and reached the maximum at 6 h of incubation. This result correlated with the COX-2 protein level which was a significant increase in the ant venom-exposed cells. It is possible that the crude venom of *T. rufonigra* triggered the production of pro-inflammatory cytokine leading to an increase in the *COX-2* expression. The mPGES-1 encodes with the mPGES-1 enzyme which is a key substance in the synthesis of inflammatory prostaglandin E2 (PGE_2_) [36]. Our study did not observe the significant upregulation of *mPGES-1* mRNA expression, whereas the PGE_2_ protein level tended to increase within 1 h and continuously elevated within 24 h after the ant venom treatment. 

The *mPGES-1* expression did not increase at the 1-h treatment, while it significantly increased about two times after the 12-h treatment indicating that the *mPGES-1* expression took a longer period after the stimulation. This may be due to the direct induction of mPGES-1 activity or the accumulation of its substrate by the ant venom. The results from this study are similar to the previous studies which have reported that the PGE_2_ level was significantly increased within 30 min and reached the maximal level at 24 h after treating the cells with LPS at 10 ng/mL in RAW 264.7 macrophage cells [19]. The COX-2 was highly expressed at 8 h and then decreased within 24 h after a treatment of melitin, the major component of honeybee venom in RAW 264.7 macrophage cells [37]. The cytosolic PLA_2_ (cPLA_2_) encoded by the *cPLA_2_* gene is an enzyme for the hydrolysis of membrane phospholipids especially phosphatidylcholine to produce lysophosphatidylcholine and arachidonic acid for initiating the inflammatory process [38]. This study found that the PLA_2_ level was not altered by the ant venom. We hypothesized that the ant phospholipase might contribute to the cleavage of phospholipid membrane of the treated cells to release arachidonic acid for inflammation activation or the other chemical substances included in the ant venom might directly activate the cPLA_2_ activity.

In conclusion, there were seven types of proteins contained in the crude *T. rufonigra* venom and exerted the phospholipase activity. The inflammatory process of the crude venom of *T. rufonigra* was directly activated through the *COX-2* and *mPGES-1* gene expression. This study only investigated the in vitro inflammatory mechanism using RAW 264.7 macrophage cells. A future study in animal models should be evaluated to provide more mechanism responses for the prevention and treatment of *T. rufonigra* venom intoxication.

## 4. Materials and Methods

### 4.1. Ant Collection and Identification

Live adults of *T. rufonigra* worker (*n* = 1875) were collected from an arboreal nest, oriental lacquer conservation project under the Royal Initiation of Her Royal Highness Princess Maha Chakri Sirindhorn (17°50′59.4′′ N 98°22′15.4′′ E), Omkoi District, Chiang Mai Province, between June and July 2018. The ants were placed in a plastic bag and frozen at −20 °C prior to dissecting the venom glands. Some ants were identified as the genus and species by the taxonomist at the Natural History Museum. Ethical standards were considered from the laboratory animal center, Chiang Mai University, Code: FA001 /2562[02/2562-01-09].

### 4.2. Venom Extraction and Quantification of Venom Protein

The venom sac of the ants were dissected from their body and subsequently soaked in phosphate buffered saline, pH 7.4. Crude venom was extracted by the mechanical method according to Pessoa et al. [12]. The suspension was centrifuged at 13,300 rpm for 15 min, and then the supernatant was collected and evaporated under nitrogen gas to obtain a protein residue. Dried protein residue was stored at −20 °C before use and was re-dissolved by ultrapure water for the experiment. The amount of total venom protein was quantitated by the BCA protein assay (Thermo Scientific, Rockford, IL, USA) following the manufacturer’s guidelines. The amount of venom protein was calculated compared with the bovine serum albumin (BSA) standard curve and expressed as μg/mL.

### 4.3. Protein Electrophoresis

Thirty micrograms of the venom protein were loaded on a 15% separating gel (40% acrylamide solution, 2% bis-acrylamide, 1.5 M Tris-HCl pH 8.8, 10% sodium dodecylsulfate (SDS), distilled water, 10% ammonium persulfate (APS) and tetramethylethylenediamine (TEMED), and 4% stacking gel (40% acrylamide solution, 2% Bis-acrylamide, 0.5 M Tris-HCl pH 6.8, 10% SDS, ddH_2_O, 10% ammonium persulfate (APS), and N,N,N′,N′-tetramethylethylene diamine (TEMED)). The gel electrophoresis was performed at 150 V for 140 min in a 1 X running buffer containing 3% *w*/*v* Tris, 14.4% *w*/*v* glycine and 10% *w*/*v* SDS. After that, the protein band in the gel was stained with a Coomassie staining solution (0.25% *w*/*v* Coomassie blue, 20% *v*/*v* methanol, and 10% *v*/*v* acetic acid) [12].

### 4.4. Protein Identification

Seven major bands of protein in the gel were cut and incubated at 37 °C until dry. Each band of protein was digested by trypsin and the digested proteins were injected to HPLC-ESI-MS/MS (AB Sciex, Les Ulis, France). Agilent Zorbax 300SB-C18, 3.5 µm (Agilent Technologies, Santa Clara, CA, USA) was used as an analytical column and coupled with 5600 Triple TOF mass spectrometers. The mobile phase was composed of 0.1% trifluoroacetic acid in water (A) and 0.09% trifluoroacetic acid in 80% acetonitrile/20% water (B). The linear gradient 25–70% of B in 40 min was used as an eluent with a flow rate of 1.0 mL/ min. Separated peptides were transferred to the mass spectrometer (5600 Triple TOF mass spectrometer) and ionized in a capillary. Next, they were fragmented in a positive mode with a selection of the minimum intensity of 10 counts, and three of the most intense ions were analyzed by each scan of 1 s, with a collision energy ranging from 20 to 95 eV according to the mass/charge (*m/z*) ratio of the peptides. The obtained spectra of the peptide were analyzed and compared with the Mascot sequence matching software (Matrix Science, Boston, MA, USA).

Three parameters were considered to confirm protein identification. The first one is the query coverage value, which was used to indicate the proportion of protein sequences aligned with the NCBI database. The value showed that more than 80% was accepted [39]. The second parameter is the expect value (E-value), which close to zero refers to a more significant match [39]. The last one is the percentage of identification. This number describes the similarity of protein sequences with the reference protein in the database. The value with more than 35% was accepted [39].

### 4.5. Secreted Phospholipases A_2_ (sPLA_2_) Activity

The sPLA_2_ activity in ant venom was measured by the egg yolk agar method with slight modification [40]. One percent (*w*/*v*) of agarose was mixed with 0.01 M of CaCl_2_ and 10% egg yolk. Then, 25 ml of the medium was poured in sterile plastic plates. After consolidation of the agar, the medium was punctured with 0.6 cm of sterile pasteur pipette. Twenty microliters of each concentration of ant venom and PLA_2_ standard (Sigma-Aldrich, St. Louis, MO, USA) were filled into the well. The plates were incubated at 50 °C for 18 h. The diameter of clear halos was measured. The sPLA_2_ activity was calculated from the PLA_2_ standard curve and expressed as µg/mg protein.

### 4.6. Cell Culture

The RAW 264.7 murine macrophages were cultured in Dulbecco’s Modified Eagle Medium supplemented with 10% fetal bovine serum and 1% antibiotic (penicillin and streptomycin) in ultra-low attachment culture dishes (Corning, Oneonta, NY, USA.) The cells were grown at 37 °C, under a humidified atmosphere of 5% CO_2_. The cells were subcultured every 2 days by vigorous pipetting over the dish surface until the cell suspended in the media. Then, the cell suspension was centrifuged at 300 g for 2 min. The supernatant was discarded, and the cell pellet was re-suspended in fresh completed media. The proper cell number and cell viability were counted by staining with trypan blue.

### 4.7. Cell Viability

RAW 264.7 cells at a density of 1 × 10^6^ cells/mL were seeded in a 96-well plate for 24 h. After incubation for 24 h, the different concentrations of venom ranging from 0.39-25 μg/mL were added into the cells. The cells were incubated for 1, 3, 6, 9, and 12 h. Then, 25 µL of the MTT solution (3-(4, 5-dimethylthiazol-2yl)-2, 5-diphenyltetrazolium bromide), (Bio Basic, Markham, ON, Canada) in PBS (5 mg/mL) was added. The cells were incubated at 37 °C, under a humidified atmosphere of 5% CO_2_ for 4 h. The formazan crystal was dissolved with dimethyl sulfoxide. The absorbance was measured at 570 and 630 nm. The percentage of cell viability was calculated according to the following formula:

% Cell viability = (absorbance of treatment − blank) / (control − blank) × 100%(1)

### 4.8. Measurement of COX-2 and PGE_2_

RAW 264.7 cells at a density of 5 × 10^5^ cells/mL were seeded in a 24-well plate and allowed to adhere overnight. After incubation for 24 h, the cells were treated with the ant venom IC_20_ concentration from the previous experiment for 1, 6, and 12 h. After that, the cells were harvested to determine COX-2 using the mouse COX-2 Simple Step ELISA Kit (Abcam, Cambridge, UK) and the supernatant was collected to determine prostaglandin E2 using the ELISA Kit (Abcam, Cambridge, UK) according to the manufacturer’s instructions.

### 4.9. Inflammatory-Released Molecule Gene Expression

To determine the inflammatory gene expression after treating the cells with the ant venom, qRT-PCR was performed. RAW 264.7 cells at a density of 5 × 10^5^ cells/mL were seeded in a 24-well plate and allowed to adhere overnight. Subsequently, total RNA was extracted using the Nucleozol reagent (Toyobo, Osaka, Japan) according to the manufacturer’s protocol. Three micrograms of the total RNA were reversed into cDNA by the RevertAid First Strand cDNA Synthesis Kit (Thermo Scientific). To determine the gene expressions, a quantitative real-time polymerase chain reaction was performed. The reaction mixture consisted of 2 µL of cDNA, 5 µL of SYBR Green, 0.04 µL of Rox reference dye, and 0.6 µL forward and reverse primers. The primers used for *cPLA_2_, COX-2, mPGES-1*, and *GAPDH* amplification are shown in Table 3. In brief, the PCR parameters for gene amplification were 40 cycles at 95 °C for 1 min for the initial denaturation, at 95 °C for 15 s for denaturation, at 60 °C for 15 s for annealing, and at 72 °C for 30 s for extension. The expression of the target mRNA level was analyzed by the 2^−∆∆CT^ method using *GAPDH* as a housekeeping gene.

All used primers were blasted with the NCBI database. The efficiency of a specific amplification of primers to target genes was evaluated by the melting curve analysis to monitor the Tm of the amplification product. Moreover, the size of the amplification products was also checked on a gel electrophoresis. The stability of the housekeeping gene was monitored by observing the same cycle time in each experiment. The PCR products were subjected to electrophoresis to confirm the product size and amplified using a specific primer.

### 4.10. Statistical Analysis

The data are presented as the mean ± SEM (standard error of the mean) of three independent experiments for each test. One-way ANOVA and Student’s t-test were used to compare between the experimental groups and the control group. A significant difference was considered at *p <* 0.05. A statistical analysis was performed with SPSS version 22 and GraphPad Prism 5.0 (GraphPad Software, Inc., San Diego, CA, USA).

## Figures and Tables

**Table 1:** Screening information of venom proteins contained in the *T. rufonigra* venom.

Band	Accession Code	Protein	Peptide Sequences	Query Cover	E-Value	% Identify
A	EFN75173.1	Paramyosin, long form	LDLER, IISKLEAR, VQLEEESEAR	100%	6 × 10^−4^	87%
	KYN11253.1	Paramyosin, short form	IDLER, IISKLEAR	100%	0.41	100%
B	XP_011143765.1	protein 5NUC	IGVIGYLTPETK, EVEDIDLVIGGHTNTFLYR, KVYVVQAYAYTK	100%	4 × 10^−10^	53%
	XP_011253192.1	Protein 5NUC	SESPSTIFLNAGDTYQGTAWYNVYK, KVYVVQAYAYTK, GDIISVLPFGNVIVK	100%	2 × 10^−10^	100%
	XP_024877450.1	protein 5NUC-like	KVYVVQAYAYTK, DDQVTRADVISVLPFGNVIVK	100%	4 × 10^−6^	82%
	*XP_025991233.1*	Protein 5NUC	EVEDIDLVIGGHTNTFLYR, KVYVVQAYAYTK, ADIISVLPFGNVIVK	100%	3 × 10^−9^	57%
C	XP_012521687.1	PREDICTED:prolyl endopeptidase-like	FLDPFLDVVTK, FLNPFLDVVTK	81%	6.3	75%
	XP_012146606.1	PREDICTED: von Willebrand factor A domain-containing protein 8 isoform X1	AVKIANTIAEIFK	100%	1.8	69%
	XP_018374341.1	PREDICTED: transcriptional activator cubitus interruptus	SGGGGGGGLGSGGSIR	87%	0.03	93%
	EZA61259.1	Aminopeptidase N	HKSLDDFSNGK	72%	4.5	88%
	OAD53823.1	Aminopeptidase N	FLGIGTLSR	72%	4.5	88%
	XP_018301168.1	PREDICTED: growth arrest and DNA damage-inducible proteins-interacting protein 1	AQYEDLAKK	88%	1.5	88%
	XP_012219589.1	PREDICTED: jmjC domain-containing protein 4 isoform X2	WVYILDGATFEVLR	100%	0.24	73%
	XP_011145278.1	ATP-binding cassette sub-family A member 3	SGMDPEK + Oxidation(M)	85%	15	100%
D	PRD20637.1	Trypsin-3	LGEDNINVVEGNEQFISASK, SIVHPSYNS NTLNNDIMLIK	97%	3 × 10^−7^	46%
	ARK19907.1	venom protein	SLDLDSIIAEVK, WELLQQVDTSTR	87%	0.024	59%
E	XP_011139048.1	protein jagged-1	LLARPLARPLALHTR	80%	0.013	91%
F	KFM72666.1	hypothetical protein X975_20223	LGEDNINVVEGNEQFISASK, SSGTSYPDVLK	83%	5.2	42%
G	KMQ91113.1	gag-pol polyprotein	SVGLDDSIR	88%	1.1	100%
	XP_024886252.1	transcription factor SPT20 homolog isoform X1	SVGLVEAER	88%	12	88%
	XP_011262712.1	phospholipase A_2_	NDGLFTR	100%	16	86%

**Table 2:** Summary of the ant venom proteins based on their function.

Group of Proteins	Type of Protein
1. The venom protein	protein 5NUC prolyl endopeptidase-like aminopeptidase N trypsin-3 venom protein phospholipase A_2_
2. The transcription activator/regulation protein	transcriptional activator cubitus interruptus
3. Cell cycle control protein	growth arrest and DNA damage-inducible proteins-interacting protein 1
4. Transporter protein	ATP-binding cassette sub-family A member 3
5. Structural protein	Paramyosin, long form Paramyosin short form
6. Ligand protein	protein jagged-1
7. hypothetical protein	-

**Table 3:** The primer sequences for the measurement of inflammatory gene expression by qRT-PCR.

Target Genes	Sequences	Reference
*cPLA_2_*	forward 5′–CAT TTA ACC TGC CAT ATC CCT-3′ Reverse- 5′–ATG GTT GGG CAA TCC TT-3′	[40]
*COX-2*	forward 5′ -GAA GTC TTT GGT CTG GTG CCT G-3′ Reverse 5′ -GTC TGC TGG TTT GGA ATA GTT GC-3′	[41]
*mPGES-1*	forward 5′- GGA TGC GCT GAA ACG TGG A- 3′ Reverse 5′- CAG GAA TGA GTA CAC GAA GCC - 3′	[42]
*GAPDH*	Forward 5′-ACC ACA GTC CAT GCC ATC AC-3′ Reverse- 5′-TCC ACC ACC CTG TTG CTG TA-3′	[42]

## Data Availability

Please refer to suggested Data Availability Statements in section “MDPI Research Data Policies” at https://www.mdpi.com/ethics.
